# Refractory Graves’ Disease With Propylthiouracil-Induced Hemophagocytic Lymphohistiocytosis and Multiple Drug Allergies Treated With Total Thyroidectomy: A Case Report

**DOI:** 10.7759/cureus.115040

**Published:** 2026-08-23

**Authors:** Kenya Kasahara, Hanako Nakajima, Hiroshi Okada, Masahide Hamaguchi, Michiaki Fukui

**Affiliations:** 1 Department of Endocrinology and Metabolism, Graduate School of Medical Science, Kyoto Prefectural University of Medicine, Kyoto, JPN; 2 Department of Diabetes and Metabolism, Osaka General Hospital of West Japan Railway Company, Osaka, JPN

**Keywords:** antithyroid drug allergy, drug lymphocyte stimulation test (dlst), graves’ disease, hemophagocytic lymphohistiocytosis (hlh), propylthiouracil (ptu)

## Abstract

Graves’ disease can be managed with antithyroid drugs (ATDs), radioactive iodine (RAI), or surgery. When ATDs are ineffective or poorly tolerated, RAI or surgery may be indicated. Methimazole (MMI) and propylthiouracil (PTU) are commonly used, sometimes with adjunctive agents such as potassium iodide (KI), β-blockers, or corticosteroids. Although ATDs are essential in management, rare but severe allergic reactions may occur, necessitating careful monitoring. We report a case of refractory Graves’ disease with a history of ATD-induced hemophagocytic syndrome and multiple drug allergies. A 43-year-old woman was diagnosed with Graves’ disease after presenting with thyrotoxicosis and was treated with MMI and KI. Following an increase in the MMI dosage, she developed a rash and was switched to PTU. Although the rash resolved, she developed fever requiring hospitalization. During admission, shock and abnormal vital signs prompted suspicion of PTU-induced hemophagocytic lymphohistiocytosis. Immunosuppressive therapy, plasma exchange, temporary hemodialysis, and corticosteroids led to improvement. She was discharged on corticosteroids, but thyroid function worsened during tapering, making ATDs unsuitable. Sixteen months later, RAI therapy was attempted; thyroid function worsened, and KI was continued. Eighteen months later, she was rehospitalized for fatigue; increasing corticosteroids improved thyroid hormone levels. Due to multiple drug allergies, a drug-induced lymphocyte stimulation test was performed preoperatively and was positive for levothyroxine (LT4), although patch and oral provocation tests were negative, confirming LT4 safety. After stabilization, total thyroidectomy was performed; postoperatively, she received powdered LT4 and calcium supplementation. The course was uneventful, without allergic manifestations, and she was discharged in good condition. This case highlights the risk of severe hematologic complications from PTU and demonstrates that, with careful preoperative evaluation and individualized perioperative management, safe surgical treatment of refractory Graves’ disease is possible.

## Introduction

Graves’ disease can be treated with antithyroid drugs (ATDs), radioactive iodine (RAI) therapy, or surgery [1]. When ATDs are ineffective or not tolerated, RAI or surgery is considered. ATDs such as methimazole (MMI) and propylthiouracil (PTU) are commonly used, often with potassium iodide (KI), β-blockers, and corticosteroids, depending on the clinical situation. Adverse events associated with ATDs include liver injury, skin rash, agranulocytosis, and antineutrophil cytoplasmic antibody (ANCA)-associated vasculitis. Although liver injury and agranulocytosis occur in approximately 0.1-0.5% of patients, they can become serious if not treated promptly [2]. Rarely, ATD-induced hypersensitivity reactions can cause severe systemic manifestations, including fever, widespread skin eruptions, lymphadenopathy, and involvement of internal organs [3]. Although ATDs are important treatments, allergic reactions, though rare, can be severe, requiring careful monitoring [4-6]. Herein, we report a case of refractory Graves’ disease with a history of ATD-induced hemophagocytic syndrome and multiple drug allergies.

## Case presentation

A 43-year-old woman, independent in activities of daily living, had hypertension, dyslipidemia, and multiple drug allergies, including amoxicillin, cefditoren pivoxil, fluvastatin, fexofenadine, and pronase. She had developed IgA nephropathy 20 years earlier, which progressed to chronic kidney disease, and autoimmune hepatitis (AIH) 13 years earlier, for which she was receiving prednisolone (PSL).

Three years before the current admission, she developed thyrotoxicosis and was diagnosed with Graves’ disease, with free triiodothyronine (FT3) 23.6 pg/mL, free thyroxine (FT4) 7.77 ng/dL, thyroid-stimulating hormone (TSH) 0.005 μIU/mL, and thyrotropin receptor antibody (TRAb) 19.6 IU/L. Thyroid ultrasonography revealed bilateral enlargement of the thyroid lobes with increased blood flow. Because the patient had severe Graves’ disease (FT4 ≥5 ng/dL), treatment with MMI 15 mg/day and KI 50 mg/day was initiated in accordance with the Japanese Guidelines for the Treatment of Graves’ Disease 2019 [7]. As the patient’s thyroid hormone levels improved and even decreased, KI was discontinued after 28 days. MMI was tapered monthly from 15 mg to 10 mg, then 5 mg, and finally 2.5 mg.

Nine months after diagnosis, her disease worsened, and the MMI dose was increased to 15 mg/day. Two weeks later, she developed a rash, which was clinically determined to be an adverse reaction to MMI, as no other medications could have caused the eruption. MMI was discontinued and replaced with PTU 300 mg/day. The rash subsequently resolved; however, fever developed eight days after starting PTU, leading to hospitalization.

She had a fever (39.4 °C), blood pressure of 120/62 mmHg, pulse rate of 122/min, and oxygen saturation of 97% on room air. The thyroid was neither tender nor enlarged. Facial flushing and a palpable cervical lymph node were noted, without rash or purpura on the extremities. Considering possible adrenal insufficiency from steroid therapy, PSL was increased from 5 mg to 20 mg. Laboratory testing showed a white blood cell count of 9,900/μL and CRP of 3.33 mg/dL; ceftriaxone was started empirically because infection could not be excluded.

Fever persisted, and on day 3, diffuse erythema appeared, raising concern for a drug allergy and prompting a switch to meropenem. On day 4, she deteriorated with hypotension (65/41 mmHg), tachycardia (133/min), purpura of the extremities, and laboratory findings of lactate dehydrogenase 1,809 U/L, aspartate aminotransferase 698 U/L, alanine aminotransferase 536 U/L, fibrin/fibrinogen degradation products 648 µg/mL, and ferritin 6,738 ng/mL (Table 1).

**Table 1:** Laboratory findings on first admission.

Parameter	Value	Unit	Reference range
Biochemical examination of blood			
Red blood cells	4.55	× 10⁶/μL	3.86-4.92
Hemoglobin	11.8	g/dL	11.6-14.8
Hematocrit	35.5	%	35.1-44.4
Mean corpuscular volume	78	fL	83.6-98.2
Platelets	4.6	× 10⁴/μL	15.8-34.8
White blood cells	6,900	/μL	3,300-8,600
Neutrophils	87.2	%	39.8-70.5
Prothrombin time-international normalized ratio	2.23	-	0.93-1.12
Activated partial thromboplastin time	143.1	seconds	23.7-34.8
Fibrinogen	99	mg/dL	207-417
Fibrin/fibrinogen degradation products	648	µg/mL	0-5
Plasmin-α2 plasmin inhibitor complex	16.8	µg/mL	0-0.9
Thrombin-antithrombin complex	>120	ng/mL	0-3.9
Soluble fibrin monomer complex	>80	µg/mL	0-6.9
Antithrombin activity	45	%	83-118
Ferritin	6,738	ng/mL	10-120
Total protein	4.1	g/dL	6.6-8.1
Albumin	2.1	g/dL	4.1-5.1
Total bilirubin	2.27	mg/dL	0.4-1.5
Aspartate aminotransferase	698	U/L	13-30
Alanine aminotransferase	536	U/L	7-23
Lactate dehydrogenase	1,809	U/L	124-222
Creatine kinase	1,644	U/L	41-153
Blood urea nitrogen	34.1	mg/dL	8-20
Creatinine	3.6	mg/dL	0.46-0.79
Sodium	130	mEq/L	138-145
Potassium	4.3	mEq/L	3.6-4.8
Chloride	100	mEq/L	101-108
CRP	14.63	mg/dL	0-0.14
Estimated glomerular filtration rate	12	mL/min/1.73 m²	-
Carcinoembryonic antigen	1.6	ng/mL	0-5
Carbohydrate antigen 19-9	17.5	U/mL	0-37
Alpha-fetoprotein	1.85	ng/mL	0-7
Cancer antigen 125	46.7	U/mL	0-35
Soluble IL-2 receptor	26,500	U/mL	157-474
Urinalysis			
pH	5.5	-	4.5-7.5
Protein	+	-	Negative
Glucose	Negative	-	Negative
Ketones	Negative	-	Negative
Red blood cells	3+	-	Negative
White blood cells	Negative	-	Negative

Adrenal function was not markedly impaired (Table 2).

**Table 2:** Hormonal assessments on first admission.

Parameter	Value	Unit	Reference range
Adrenocorticotropic hormone	5.76	pg/mL	7.2-63.3
Cortisol	8.47	µg/dL	6.24-18
Thyroid-stimulating hormone	0.005	μIU/mL	0.61-4.23
Free triiodothyronine	2.12	pg/mL	2.4-4
Free thyroxine	2.08	ng/dL	0.9-1.7
Thyrotropin receptor antibody	10.9	IU/L	0-1.9

PTU was discontinued, and she was transferred to the intensive care unit with cytokine storm, disseminated intravascular coagulation, and multiorgan failure.

Noncontrast chest and abdominal CT showed splenomegaly without an infectious source. Blood and urine cultures were negative; the infectious workup was unremarkable; no significant elevations in tumor markers were detected; and autoimmune antibody testing was also negative (Tables 3, 4).

**Table 3:** Antibody test on first admission.

Parameter	Value	Unit	Reference range
Antinuclear antibody	<40	times	0-39
Proteinase 3 antineutrophil cytoplasmic antibody	<1.0	U/mL	0-3.4
Myeloperoxidase antineutrophil cytoplasmic antibody	<1.0	U/mL	0-3.4
Anti-SS-A antibody	<1.0	U/mL	0-9.9
Anti-SS-B antibody	<1.0	U/mL	0-9.9
Anti-aminoacyl tRNA synthetase antibody	<5.0	INDEX	0-24.9
Anti-melanoma differentiation-associated gene 5 antibody	<4.0	INDEX	0-31
Anti-double-stranded DNA antibody	<10	IU/mL	0-12
Anti-Sm antibody	<1.0	U/mL	0-9.9

**Table 4:** Infection test on first admission.

Parameter	Value	Unit	Reference range
Interferon-gamma release assay	Negative	-	Negative
β-D-glucan	7.2	pg/mL	0-20
Epstein-Barr virus DNA	2.7	Log IU/mL	0
Epstein-Barr virus nuclear antigen antibody	10	times	0-9
Epstein-Barr virus viral capsid antigen IgM antibody	<10	times	0-9
Epstein-Barr virus viral capsid antigen IgG antibody	20	times	0-9
C7-HRP antigenemia assay	Negative	-	Negative
Cytomegalovirus IgM antibody	0.57	INDEX	0-0.84
Cytomegalovirus IgG antibody	195	AU/mL	0-5.9
Hepatitis A virus IgM antibody	0.4	S/CO	0-0.79
Hepatitis B core antibody	0.07	S/CO	0-0.99
Hepatitis B surface antigen	≤0.04	IU/mL	0-0.04
Hepatitis B surface antibody	<1.61	mIU/mL	0-9.99
Hepatitis C virus antibody	0.08	S/CO	0-0.99
Serological tests for syphilis	Negative	-	Negative
Treponema pallidum antibody	Negative	-	Negative
Human herpesvirus 6 DNA	<20	copies/10⁶ cells	<20
HIV antigen/antibody	Negative	-	Negative

**Figure 1:**
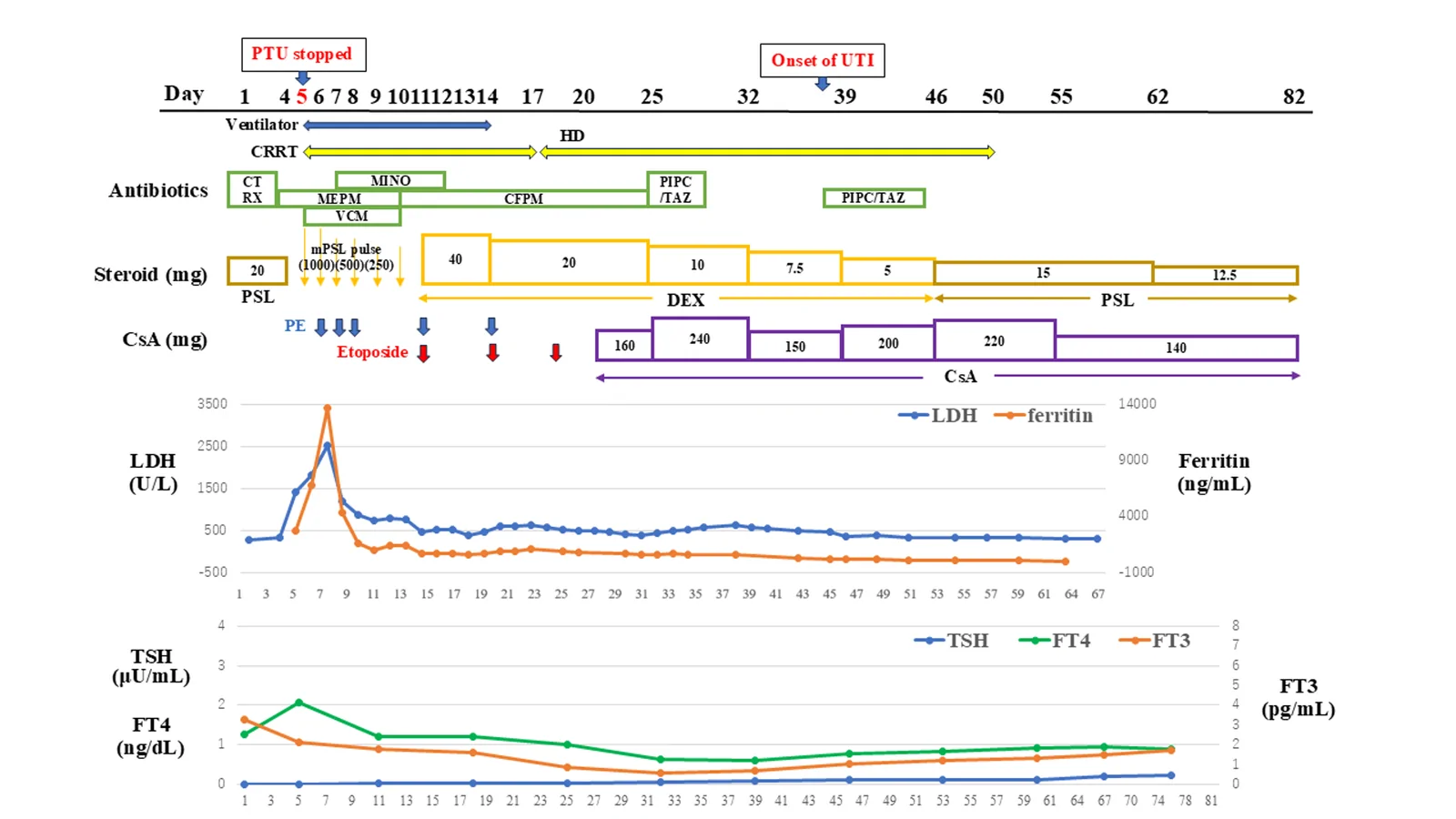
Clinical course after the first admission. CFPM, cefepime; CRRT, continuous renal replacement therapy; CsA, cyclosporine; CTRX, ceftriaxone; DEX, dexamethasone; FT3, free triiodothyronine; FT4, free thyroxine; HD, hemodialysis; MEPM, meropenem; MINO, minocycline; mPSL, methylprednisolone; PE, plasma exchange; PIPC/TAZ, piperacillin/tazobactam; PSL, prednisolone; PTU, propylthiouracil; TSH, thyroid-stimulating hormone; UTI, urinary tract infection; VCM, vancomycin

Fluorodeoxyglucose PET was performed, but no abnormal areas of increased uptake were identified. Bone marrow biopsy demonstrated hemophagocytosis. Although drug-induced allergy due to ceftriaxone or MMI was considered, the drug-induced lymphocyte stimulation test (DLST) was negative for both. After switching from MMI to PTU for a rash, the skin symptoms resolved, suggesting PTU as the more likely causative agent. She subsequently developed high fever (≥38.5 °C), splenomegaly, elevated soluble IL-2 receptor levels, and hyperferritinemia, leading to the diagnosis of PTU-induced hemophagocytic lymphohistiocytosis (HLH) according to the HLH-2004 diagnostic criteria [8]. Temporary hemodialysis, mechanical ventilation, and plasma exchange were performed. She was treated according to the HLH-2004 protocol with etoposide, cyclosporine A, and corticosteroids (Figure 1).

Etoposide 120 mg was administered on days 10, 13, and 17, and cyclosporine was initiated on day 20 after renal function showed a trend toward improvement [8]. Her condition gradually improved, and she was discharged on PSL monotherapy with ATDs discontinued as TRAb became negative, which might have been influenced by immunosuppression due to steroids and the effects of plasma exchange.

Following discharge, tapering of PSL resulted in recurrent hyperthyroidism. Three months before the second admission, KI 50 mg was reinitiated due to FT3 12.8 pg/mL, FT4 4.3 ng/dL, and TSH 0.006 μIU/mL. Thyroid function improved within a month (FT3 3.54 pg/mL, FT4 1.56 ng/dL, and TSH 0.006 μIU/mL). As ATDs were difficult to use, RAI was considered. Due to the need for a two-day iodine restriction, which was considered insufficient yet acceptable given the plan for potential repeated RAI treatments and to avoid rapid hyperthyroidism from abrupt KI discontinuation, the I-131 dose was set at 10 mCi, considering reduced iodine uptake, body weight (63 kg), and renal impairment (serum creatinine of 1.92 mg/dL and estimated glomerular filtration rate of 23.6 mL/min/1.73 m²). Although this therapy was given two months before the second admission, thyroid hormone levels actually worsened. About a month before the second admission, KI 50 mg/day was resumed without effect, and two weeks before the second admission, the dose was increased to 100 mg/day with PSL 6 mg/day for AIH. However, she was rehospitalized due to worsening fatigue.

**Figure 2:**
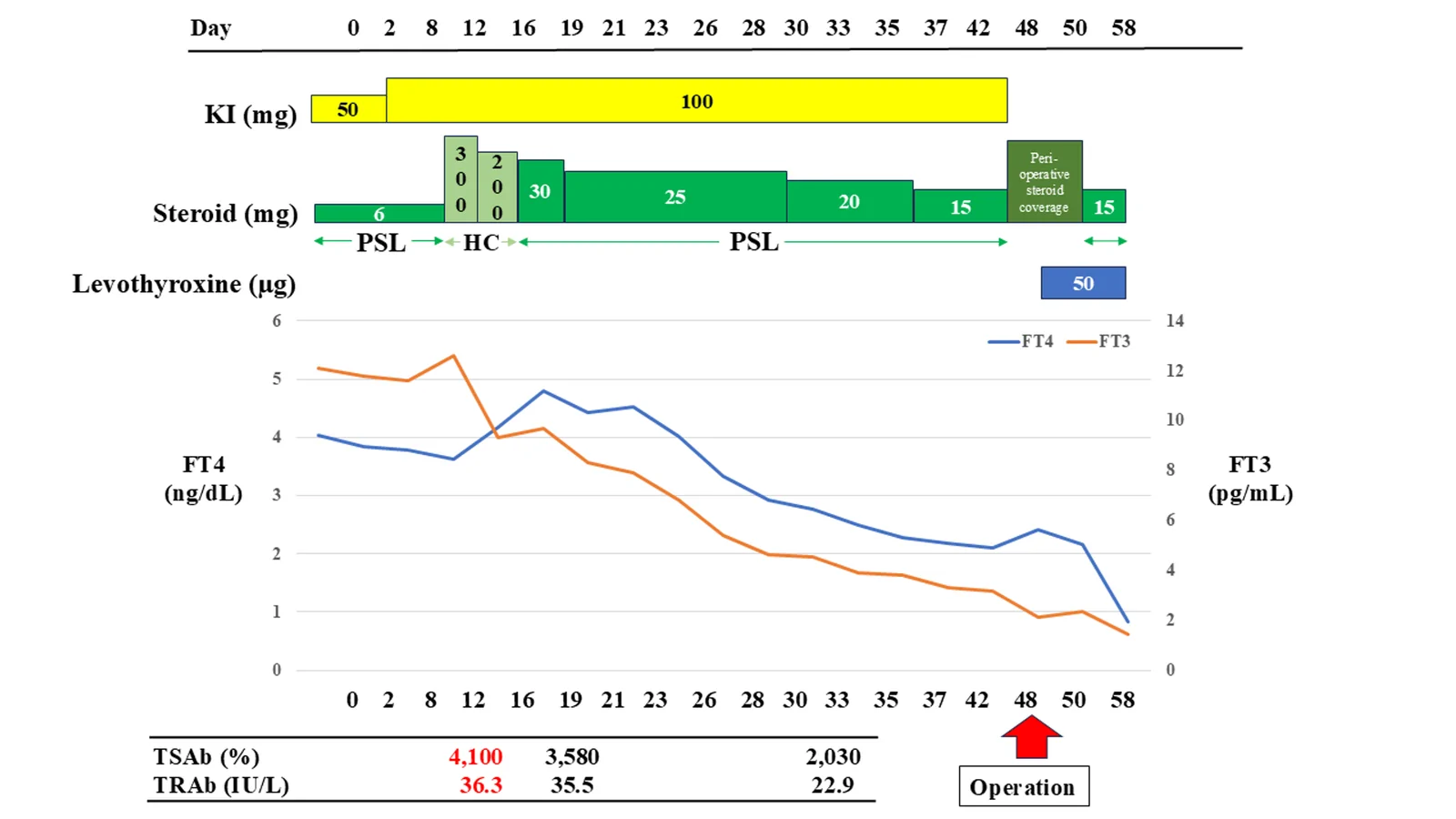
Clinical course after the second admission. FT3, free triiodothyronine; FT4, free thyroxine; HC, hydrocortisone; KI, potassium iodide; TRAb, thyrotropin receptor antibody; TSAb, thyroid-stimulating antibody

On admission, medications included KI 100 mg/day, esomeprazole 20 mg/day, alendronate 35 mg/week, fluconazole 200 mg/day, PSL 6 mg/day, olmesartan 40 mg/day, febuxostat 10 mg/day, and ferrous fumarate 50 mg/day. She was afebrile (37.0 °C), with blood pressure of 113/65 mmHg, pulse rate of 99/min, and oxygen saturation of 98% on room air. Physical examination showed mild thyroid enlargement without tenderness or exophthalmos. Laboratory testing showed FT3 11.6 pg/mL, FT4 3.79 ng/dL, and TSH 0.005 µIU/mL. Ultrasonography showed bilateral thyroid enlargement (right 24 cm³, left 11.8 cm³), increased vascularity, heterogeneous echotexture, and peak systolic velocity of 43.5 m/s in the right superior thyroid artery. Given her multiple drug allergies and limited response to medical and RAI therapy, which may have triggered destructive thyroiditis, total thyroidectomy was considered. Despite increased KI, thyroid function remained elevated (FT3 12.6 pg/mL, FT4 3.63 ng/dL). Hydrocortisone was started on day 9 to support thyroid hormone management in the setting of high disease activity (thyroid-stimulating antibody (TSAb) 4,110%, TRAb 36.3 IU/L). On day 15, PSL was increased to 30 mg/day, gradually improving thyroid hormone levels. By day 42, FT3 decreased to 3.19 pg/mL and FT4 to 2.1 ng/dL, with improving antibody levels; PSL was tapered to 15 mg/day (Figure 2).

Preoperatively, DLST was performed to assess perioperative medications. Levothyroxine (LT4) (powder and tablet) was DLST-positive; however, both patch testing and oral provocation testing with LT4 (powder) were negative (Table 5).

**Table 5:** Drug-induced lymphocyte stimulation test.

Parameter	Maximum stimulation index	Result	Reference range
Levothyroxine sodium hydrate (tablet)	2.5	+	0-1.7
Levothyroxine sodium hydrate (powder)	2.1	+	0-1.7

Given the prior LT4 tablet-associated allergic symptoms, an oral challenge with LT4 powder was performed and was well tolerated.

**Figure 3:**
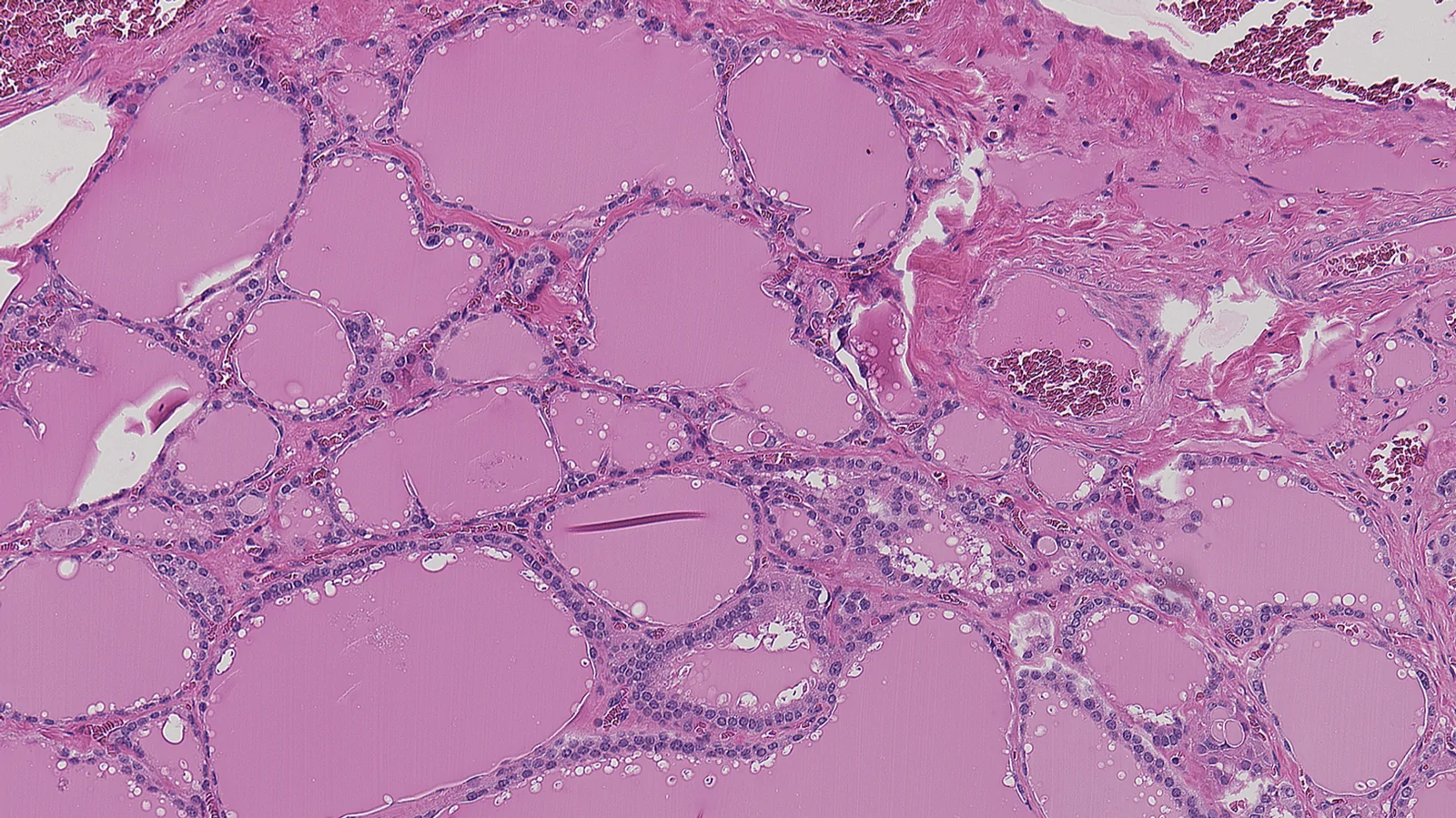
Histopathological findings of the resected thyroid (hematoxylin and eosin staining, original magnification ×100). Representative section showing diffuse follicular hyperplasia with papillary infolding and mild stromal lymphocytic infiltration, consistent with Graves’ disease.

Seven weeks later, total thyroidectomy was performed with perioperative steroid coverage. Pathology showed diffuse thyroid enlargement with epithelial cells without nuclear atypia forming follicles, papillary infolding into the lumen, and mild stromal lymphocytic infiltration, consistent with diffuse hyperplasia due to Graves’ disease (Figure 3).

Postoperatively, the patient continued LT4 powder without any allergic manifestations. PSL 15 mg/day was continued for both AIH and postoperative management, and she was discharged on postoperative day 6, with no allergic symptoms during the postoperative course or after discharge.

## Discussion

This case involved a patient with PTU-induced HLH and multiple drug allergies who ultimately underwent total thyroidectomy. HLH, characterized by activated macrophages phagocytosing autologous blood cells in the bone marrow, spleen, and lymph nodes, presents with fever, cytopenia, coagulopathy, and liver dysfunction and may be fatal [8]. In adults, HLH is usually secondary to viral infections, malignancies, or collagen vascular diseases; however, none were identified in this case [9]. In this case, there were no significant findings suggestive of infection or malignancy, and the patient fulfilled the HLH-2004 diagnostic criteria; therefore, in the absence of other identifiable causes, drug-induced HLH was diagnosed by exclusion. As no other medications were considered potential causes, the condition was diagnosed as PTU-induced HLH. This diagnosis was further supported by the clinical improvement observed after discontinuation of PTU and subsequent treatment. Although HLH with PTU-induced ANCA-associated vasculitis has been reported, no prior cases directly linking PTU to HLH have been reported, making this case noteworthy [10].

In this patient, multiple drug allergies limited ATD use, and RAI, which requires thyroid destruction and lacks an immediate corrective effect, was challenging, making surgery the preferred option. For perioperative risk reduction, strict preoperative control of thyrotoxicosis is crucial, and when ATDs are unavailable, KI can help control hyperthyroidism by inhibiting thyroid hormone release and decreasing thyroid blood flow [11]. In this case, KI alone was insufficient; however, adding corticosteroids, following a thyroid storm protocol, achieved biochemical improvement. Some reports suggest that KI plus corticosteroids lowers FT3 more effectively than KI monotherapy [12]. Although corticosteroids are not standard for Graves’ disease, they are sometimes used temporarily in thyroid eye disease or thyroid storm [13-15]. Here, corticosteroids were administered preoperatively to suppress thyroid hormone levels and TRAb, representing a deviation from standard management, and should be viewed as an adjunctive measure under special circumstances [13].

Perioperative drug selection was difficult given the patient’s extensive allergy history. Although severe ATD allergy is rare, corticosteroids are required in such severe cases [2]. Only a few PTU-induced drug-induced hypersensitivity syndrome (DIHS) cases have been reported [3,16]. DIHS shares clinical features with HLH, and T-cell-driven cytokine elevation can activate macrophages and cause hemophagocytosis, suggesting a similar mechanism [17]. Although cutaneous manifestations here were atypical of DIHS, immune activation leading to organ damage may have acted via a similar mechanism. Given her severe course of drug-induced HLH, drug selection was cautious. Although DLST has many false positives, it remains part of the evaluation, with patch and oral challenge tests often needed for confirmation [18]. In this case, DLST for LT4 was positive, but both patch and oral challenge tests were negative, possibly influenced by concurrent corticosteroids, and postoperative LT4 was well tolerated without allergic reactions.

## Conclusions

We report a rare case of refractory Graves’ disease complicated by PTU-induced HLH and multiple drug allergies, challenging conventional therapy. Despite limited options, total thyroidectomy was successfully performed after careful preoperative optimization, allergy testing, and adjunctive corticosteroid therapy. This case highlights the risk of severe hematologic complications from PTU and demonstrates that individualized management can achieve favorable outcomes even in challenging clinical scenarios.
